# Identification of Specific Anaerobic Bacteria in Endodontic Infections of Primary Teeth—A PCR Study

**DOI:** 10.5005/jp-journals-10005-1573

**Published:** 2019

**Authors:** Umapathy Thimmegowda, Joseph Thomas, Shivaprasad Bilichodmath, Naveena Preethi

**Affiliations:** 1,2,4 Department of Pedodontics and Preventive Dentistry, RajaRajeswari Dental College and Hospital, Bengaluru, Karnataka, India; 3 Department of Pedodontics and Preventive Dentistry, Krishnadevaraya College of Dental Sciences and Hospital, Bengaluru, Karnataka, India

**Keywords:** Anaerobic, Endodontic infection, PCR, Primary dentition

## Abstract

**Introduction:**

Invasion of microorganisms and their multiplication in root canals (RCs) results in endodontic infections of primary teeth. Acute and chronic inflammation may be present in the periapical area and are based on the amount and virulence of microorganisms, especially anaerobic bacteria present in the RC. To identify microorganisms very precisely in endodontic infections, polymerase chain reaction (PCR) is used.

**Aim:**

The aim of the present study is to identify the specific anaerobic bacteria like *Porphyromonas gingivalis*, *Prevotella intermedia*, and *Actinomyces naeslundii* in the RCs of primary teeth using real-time PCR.

**Methodology:**

Fifteen subjects aged 3–8 years who had endodontic infections in primary molars were selected. The cases who had been selected did not receive any endodontic treatment and antibiotics within 3 months, and children with systemic diseases were not included.

**Sample collection:**

Samples were taken by placing absorbent paper points into the largest canals of maxillary and mandibular molars for 60 seconds and are then transferred to a sterile Eppendorf tube with tris-hydochloride EDTA (TE) buffer. The samples were stored at −80°C. All samples were subjected to PCR analysis.

**Result:**

The specific anaerobes detected in the samples were *A. naeslundii* (93.3%), *Prevotella intermedia* (53.3%), and *Porphyromonas gingivalis* (13.3%).

**Conclusion:**

The results suggested a high bacterial diversity in the RCs of infected primary teeth.

**How to cite this article:**

Thimmegowda U, Thomas J, *et al.* Identification of Specific Anaerobic Bacteria in Endodontic Infections of Primary Teeth—A PCR Study. Int J Clin Pediatr Dent 2019;12(1):1–4.

## INTRODUCTION

Infections of pulp in the deciduous tooth are mainly due to the entry of bacteria and amplification in the RCs and pulp chamber. Infections in teeth are inflammatory diseases of the RC system caused by microbial infections. Acute or chronic infection is established in the RC or periradicular area based on the microbiology and nature of organism present.

The species most frequently found in the pulp chamber of primary teeth are *Porphyromonas gingivalis* (73.3%), *Prevotella intermedia* (6.7%), *Porphyromonas nigrescens* (86.7%), and *Fusobacterium alocis* (73.3%). Frequently found microbial species in RCs of primary teeth were *Porphyromonas gingivalis* (100%), *Prevotella intermedia* (6.7%), *Actinomycosis naeslundii* (7.1%), *Porphyromonas nigrescens* (93.3%), and *Treponema forsythia* (26.7%).^1^

Precise identification and culture of many anaerobic microorganisms are difficult. Molecular genetics methods have been used recently in endodontic infections, for the identification of microorganisms. Advancement in molecular genetics led to the detection of microbes directly in clinical samples. PCR a molecular procedure has been widely used in the identification of various microorganisms. PCR assays are sensitive and identification of microbes or strains that are even impossible to culture can be identified very precisely.^2^

Diagnosis of human pathogens by the real-time PCR technique has reformed the clinical microbiological laboratories. This advanced technique combines PCR chemistry with the fluorescent probe in the detection of an amplified product in the same reaction vessel in an hour or less, which is considerably faster than conventional PCR methods. So this technology was referred to as rapid-cycle real-time PCR.^3^ Rapid analysis, precision, ease of quantification, better control of quality in the process, greater sensitivity, reproducibility, and lower risk of contamination are some of the advantages of real-time PCR.^4^ Dentist should know about the normal microbiology of the oral cavity so that appropriate antibiotics can be used to eliminate these organisms. However, few researches have been performed using molecular genetics for the identification of microorganisms in primary tooth with necrotic pulp and periapical pathology.

The purpose of this study is to detect and identify the specific microorganisms like *Porphyromonas gingivalis*, *Prevotella intermedia*, and *Actinomyces naeslundii* in the RCs of human deciduous molars with endodontic infections using real-time PCR.

## METHODOLOGY

Fifteen subjects of age ranged from 3 to 8 years who reported to the Department of Pedodontics and Preventive Dentistry with pulp infections in primary molars were randomly selected. A detailed medical and dental history was taken. Children showing clinical and radiographic signs of necrotic pulp in primary molars, intact roots, resorption less than one-third of the physiologic root, and negative history of trauma to the teeth were included. Children who have received antibiotic therapy in the last 3 months and history of any systemic disease were excluded from the study.

## CLINICAL PROCEDURES

Sample collection and clinical procedures were adapted and accommodated from Jacinto et al.^5^ Local anesthesia was given, and asepsis of the oral cavity was achieved with 0.12% chlorhexidine gluconate. The concerned tooth was polished coronally and isolated with a rubber dam. The tooth, clamp, and rubber dam were also disinfected with sterile swabs soaked in 30% hydrogen peroxide followed by 2.5% sodium hypochlorite for 30 seconds each, and then were neutralized with a sterile 5% sodium thiosulfate solution. Caries was removed using sterile burs, and access preparation was carried out with an Endo access bur at a high speed under irrigation with sterile 0.9% (w/v) sodium chloride and a coronal access was gained.

## SAMPLE COLLECTION

**Fig. 1:**
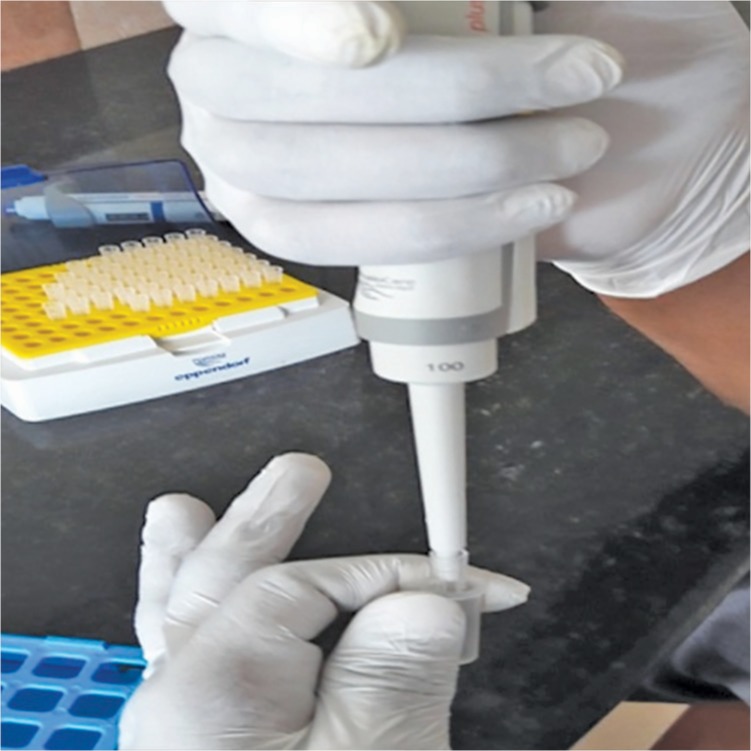
TE buffer in the Eppendorf tube

**Fig. 2:**
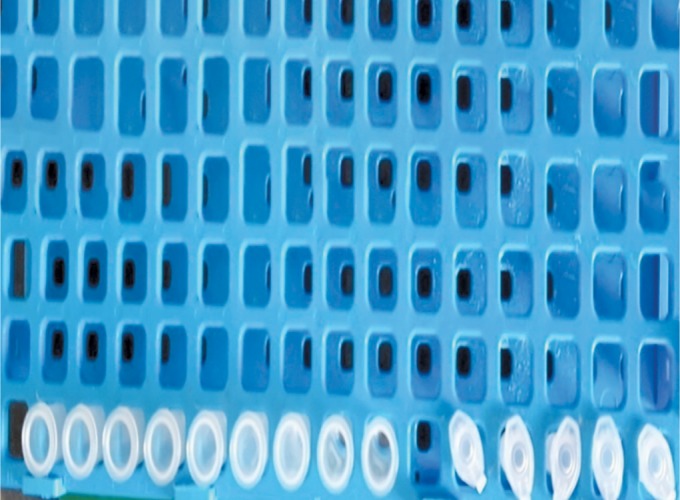
Samples collected in the Eppendorf tube and stored at −80°C

Once the access opening was gained, samples were taken from the largest RC of a concerned primary molar tooth. A sterile absorbent paper point was placed into the full length of the largest canal for 60 seconds and then was transferred to a sterile Eppendorf tube with a buffer (Fig. 1). In the case of the dry canal, the paper point was moistened in a sterile saline solution. All the collected samples were stored at −80°C (Fig. 2). Once the sample collection was done, all primary molars involved were pulpally treated, obturated, and finally restored.

## IDENTIFICATION OF SPECIFIC SPECIES BY PCR

The PCR procedure was carried out in the laboratory for molecular biology at RajaRajeswari Dental College and Hospital. Analysis of *Prevotella intermedia*, *Porphyromonas gingivalis*, and *A. naeslundii* was done using real-time PCR. DNA was extracted from the necrosed RC samples using highly purified Invitrogen DNA isolation kit (Purelink™ DNA extraction kit, Applied Biosystems, India). The bacterial primers used were Custom SYBR^®^ Green assay reagents (Applied Biosystems, India) and the primer sequences are given in Table 1.

**Table 1:** Primer pair organism and the bacterial primer

*Primer pair of organism*	*Sequence*
*Prevotella intermedia*	Forward primer
	3’-TGCAACTTGCCTTACAGAGGG-5’
	Reverse primer
	5’-ACTCGTATCGCCCGTTATTC-3’
*Porphyromonas gingivalis*	Forward primer
	3’-TGCAACTTGCCTTACAGAGGG-5’
	Reverse primer
	5’-ACTCGTATCGCCCGTTATTC-3’
*Actinomyces naeslundii*	Forward primer
	3’-ACAGCAATGCACTCCCTCAA-5’
	Reverse primer
	5’-TGCTTGGCAACGTGACGGC-3’

### DNA Extraction

Before DNA extraction, the paper points were placed in the TE buffer at the room temperature for 2 hours. The vials were centrifuged for 2 minutes at 3,000 rpm. A sterile microcentrifuge tube was taken and 20 μL of the solution was transferred and 20 μL of proteinase K was added. Occasional vortexing was done to the tubes that were kept at 55°C for 2 hours in a water bath. In the lysate, 20 μL of RNAse-A was added and was mixed well by vortexing it briefly and incubated at the room temperature for 2 minutes. The Purelink™ genomic lysis/binding buffer was added then and mixed well by vortexing to have a homogenous solution. About 96% to 100% ethanol of 200 μL was then added and mixed well by vortexing. Then the obtained homogenous solution was followed and subjected to purification.

### DNA Purification

Genomic DNA purification was done using the spin column-based centrifugation method for 10 to 15 minutes. The lysate prepared with the Purelink™ genomic lysis/binding buffer and ethanol was allotted to the spin column and centrifuged at 1,000 rpm for 1 minute at the room temperature and the column was placed in the pure link collect tube.

**Fig. 3:**
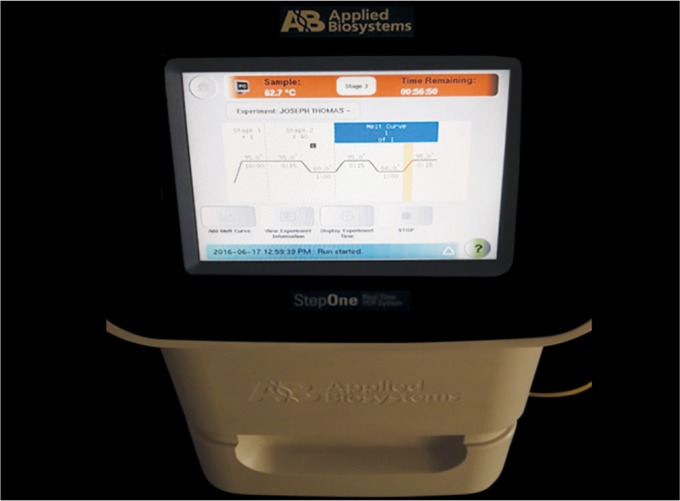
Real-time PCR machine running the protocol for species evaluation

### Wash Buffer

500 μL of wash buffer 1 and 2 were added sequentially to the spin column and centrifuged for 3 minutes at a maximum speed in in the room temperature. The spin column was placed in a sterile 1.5-mL microcentrifuge tube to which 40 μL of Purelink™ genomic elution buffer was added and incubated at the room temperature for 1 minute. The column was centrifuged for 1 minute at the room temperature, thus, obtaining purified genome DNA and was stored at −20°C.

### PCR Protocol

The PCR protocol composed of a reaction solution of SYBR® Green Universal PCR Master Mix (10 μL), forward primer (1 μL), and reverse primer (1 μL) for the specific organism, extracted DNA of unknown sample (3 μL), and nucleus-free water to make a complete reaction volume of 20 μL. The working conditions for real-time PCR were the following: the holding stage at 95°C for 10 seconds followed by 40 cycles of shuttle heating at 95°C for 15 seconds and at 60°C for 1 minute. The melt curve stage was at 95°C for 15 seconds, 60°C for 1 minute, and 95°C for 15 seconds (SYBR® Green assay reagents, Applied Biosystems, India) (Fig. 3). The PCR protocol was carried out and calculations were made. All the calculations were done using the applied biosystems software.

## RESULTS

All the samples collected showed positive for bacterial DNA when the primers directed to the specific microorganism were used. The study data were subjected to statistical analysis. The present study showed the following percentage of specific anaerobic bacteria: *A. naeslundii* (93.3%), *Prevotella intermedia* (53.3%), and *Porphyromonas gingivalis* (13.3%). The frequency distribution for the prevalence of different bacterial species was expressed in terms of number and percentage. The Chi-square Goodness of Fit test was used to compare the distribution of different types of bacterial species isolated from the necrotic RCs of primary molars.

**Table 2:** Comparison of the distribution of different bacterial species isolated from the RC of primary teeth using Chi square Goodness of Fit test

*Bacterial species*	*Category*	*n*	*%*	*χ^2^ value*	*p value*
*Prevotella intermedia*	Absent	7	46.70	0.067	0.80
	Present	8	53.30		
*Porphyromonas gingivalis*	Absent	13	86.70	8.067	0.005*
	Present	2	13.30		
*Actinomyces neaslundii*	Absent	1	6.70	11.267	0.001*
	Present	14	93.30		

* Statistically significant

Table 2 shows a comparison of the distribution of different bacterial species isolated from the RCs of primary teeth using the Chi-square Goodness of Fit test. Out of 15 samples, 8 showed the presence of *P. intermedia*, and *Porphyromonas gingivalis* were present only in 2 samples, while *A. naeslundii* were found in 14 samples. The detection of *A. naeslundii* and *P. gingivalis* in the RC was statistically significant.

**Fig. 4:**
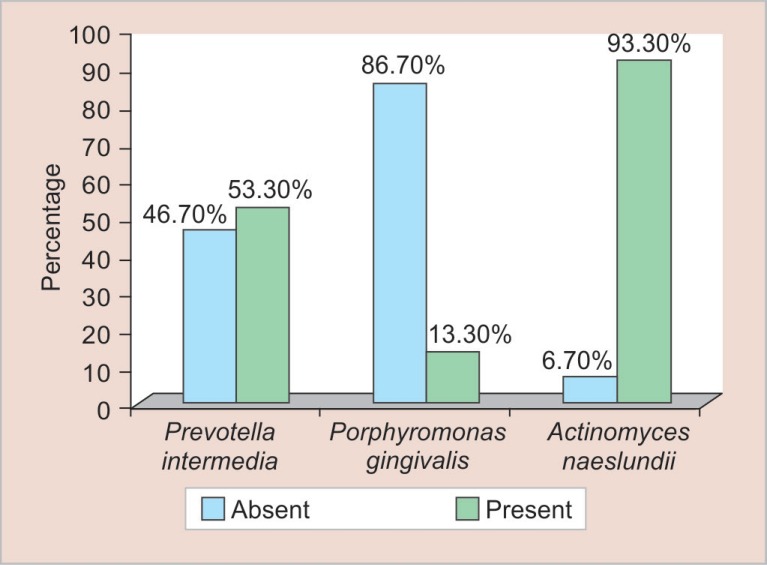
Graphical representation of percentage of different bacterial species isolated from primary teeth

Figure 4 shows a graphical representation of the percentage of three anaerobic microorganisms present in the RCs of infected primary teeth.

## DISCUSSION

Endodontic infections of primary teeth are mixed, and semi-specific with a great predominance of obligate and facultative anaerobic bacteria. There are only a few studies done on RC microbiology of deciduous teeth. Bacterial species in RC infections were detected using microbiological culture and molecular methods. Bacterial culture has played a key role in the identification of predominant and specific bacteria causing endodontic infections. Different methods involved in the detection and identification of microorganisms in endodontic microbiota are DNA–DNA hybridization, denaturing gradient gel electrophoresis fingerprint, polymerase chain reaction (PCR), or DNA sequencing techniques.^2^ The present study focuses on the detection of anaerobic microorganism associated with endodontic infection of deciduous dentition using molecular genetics, particularly polymerase chain reaction which is more specific and sensitive in detecting microorganisms. Tavares et al. detected *Prevotella intermedia* in 96.9% of the samples with pulp necrosis.^6^ About 53.3% of the sample in our study showed the presence of *Prevotella intermedia* and 13.3% of *Porphyromonas gingivalis* which is in accordance with the study done by Yang et al.^7^ Cao et al. found that *Porphyromonas gingivalis* (33%) and *Prevotella intermedia* (45%) are present in the RC associated with primary endodontic infection which is similar to our result.^8^ Topcuoglu et al. detected *Prevotella intermedia* (86%) as the prevalent microorganism in primary tooth endodontic infection.^9^ A PCR study done by Gomes et al. showed *Porphyromonas gingivalis* (100%) and only 6.7% of *Prevotella intermedia* in primary teeth exhibiting periapical radiolucency.^1^ Cogulu et al. reported 16% of *Porphyromonas gingivalis* in deciduous RC which is in accordance with our study.^2^ Higher frequency of *A. naeslundii* was detected in the present study than revealed by Kutlovci et al.,^10^ Tang et al., attributed the result to the criteria for sample selection, method of sample collection, processing, and method of detection.^11^ The discrepancies may also be due to geographic differences and manual dexterity. Actinomyces species have been recovered from various infected RCs and periapical lesions and a possible mechanism could be from periodontium to pulp chamber. Actinomyces species grown in the laboratory is slow and unpredictable, identification by biochemical technique is also unreliable. Real-time PCR technique was used in the present study to overcome these disadvantages. Tang et al. show 9.4% of *A. naeslundii* in a study done on infected RCs of the Chinese population and were found to be associated with permanent traumatized teeth.^11^ Only a few studies in the literature have done to detect the presence of *A. naeslundi* in infected primary teeth. Siqueira et al. stated that the factors included like the quality of water supply, feeding habits, access, utilization of dental health care, and socioeconomic conditions resulted in the geographical difference of distribution of bacteria.^12^ The incidences of the microorganisms detected in the present report were different as compared with other studies suggesting that the microbial composition of endodontic infections of primary teeth was heterogeneous. Identification of specific microorganisms helps us to formulate a treatment plan which includes cleaning and shaping of RCs under proper medication. The reduction and expulsion of bacterial infection of primary teeth depends on the cleaning and shaping of RC. The success of endodontic treatment in pediatric dentistry confides in the neutralization of necrotic content, instrumentation, intracanal dressings, and proper antibiotic therapy.

## CONCLUSION

The presence of anaerobic bacteria and its effect on the pathogenesis of RC is undeniable. Strict anaerobes are usually found in the RCs of infected primary teeth. The present study also confirms the presence of diverse and multifarious types of anaerobic microorganism in the necrosed RCs of human primary teeth. The identification of these specific microorganisms helps us to formulate a proper treatment plan, which includes biomechanical preparation of RCs under proper medication. The more extensive study should be done to establish the presence of these bacterias in RCs of primary teeth.
